# Geochemical and Microbial Community Attributes in Relation to Hyporheic Zone Geological Facies

**DOI:** 10.1038/s41598-017-12275-w

**Published:** 2017-09-20

**Authors:** Z. Hou, W. C. Nelson, J. C. Stegen, C. J. Murray, E. Arntzen, A. R. Crump, D. W. Kennedy, M. C. Perkins, T. D. Scheibe, J. K. Fredrickson, J. M. Zachara

**Affiliations:** 1 Energy and Environment Directorate, Pacific Northwest National Laboratory, Richland, WA USA; 2 Environmental and Biological Sciences Directorate, Pacific Northwest National Laboratory, Richland, WA USA; 3 Graphic Design, Pacific Northwest National Laboratory, Richland, WA USA; 4 Environmental Molecular Sciences Laboratory, Pacific Northwest National Laboratory, Richland, WA USA; 5 Physical and Computational Sciences Directorate, Pacific Northwest National Laboratory, Richland, WA USA

## Abstract

The hyporheic zone (HZ) is the active ecotone between the surface stream and groundwater, where exchanges of nutrients and organic carbon have been shown to stimulate microbial activity and transformations of carbon and nitrogen. To examine the relationship between sediment texture, biogeochemistry, and biological activity in the Columbia River HZ, the grain size distributions for sediment samples were characterized to define geological facies, and the relationships among physical properties of the facies, physicochemical attributes of the local environment, and the structure and activity of associated microbial communities were examined. Mud and sand content and the presence of microbial heterotrophic and nitrifying communities partially explained the variability in many biogeochemical attributes such as C:N ratio and %TOC. Microbial community analysis revealed a high relative abundance of putative ammonia-oxidizing Thaumarchaeota and nitrite-oxidizing Nitrospirae. Network analysis showed negative relationships between sets of co-varying organisms and sand and mud contents, and positive relationships with total organic carbon. Our results indicate grain size distribution is a good predictor of biogeochemical properties, and that subsets of the overall microbial community respond to different sediment texture. Relationships between facies and hydrobiogeochemical properties enable facies-based conditional simulation/mapping of these properties to inform multiscale modeling of hyporheic exchange and biogeochemical processes.

## Introduction

The hyporheic zone (HZ) plays a major role in macronutrient (C, N, P) cycling in rivers^1–3^. Within this permanently saturated transition zone between a surface channel and the surrounding banks and underlying sediments^4^, hydraulic exchange creates strong vertical, longitudinal, and lateral hydrologic and biogeochemical gradients (e.g., DO, ammonium, nitrate, DOC)^5–8^ and mediates transport of nutrients between the surface and subsurface^9^. The confluence of nutrients in the HZ creates a biogeochemical ‘hot spot’^10^, stimulating microbial activity such that it has been observed to perform up to 90% of lotic respiration^11^. In addition, both nitrification and respiratory denitrification activity have been observed within the HZ^12^.

The composition of microbial communities in the HZ are still largely unexplored. While some work has been done characterizing the dynamics of microbial communities in the hyporheic in response to environmental conditions, much of it has been done using denaturing gradient gel electrophoresis (DGGE)^13,14^, fluorescent *in situ* hybridization (FISH)^15^ or terminal restriction fragment length polymorphism (T-RFLP)^14,16–18^. These techniques reveal changes in composition but don’t generate comprehensive information about the taxonomy of the membership. A few studies have performed amplicon sequencing, however, some used bacteria-specific primers^19,20^, leaving the archaeal component of the community undescribed, while the others focused on human impact factors such as land use^21^ and metal contamination^22^.

Microbial community composition is sensitive to changes in physical and biogeochemical factors and shifts in composition can influence ecosystem processes^23^. Sediment permeability, water chemistry, and particulate organic matter appear to be key factors controlling microbial structure and activity in riverine environments^24–27^. Grain size distribution in sediment beds governs permeability^28,29^ and thus, in the HZ, affects the hydraulic linkage with the surface channel and the supply of resources (e.g., nutrients)^1,9,30^. For example, grain size has been shown to influence organic matter accumulation, microbial abundance and activity, and interstitial dissolved O_2_ concentrations^26,31^. Increased fine particle content in the HZ tends to promote higher microbial abundances in hyporheic sediments, presumably by increasing the surface area available for colonization^32–34^. Associated increases in metabolic activity and limited recharge rates due to low permeability result in sub-oxic or anaerobic conditions^26,31,34,35^. Thus there appears to be dynamic feedback between local physicochemistry and microbial community structure and function that is governed by sedimentary structure.

Facies are elements of a sediment classification scheme that group complex geologic materials into a manageable set of discrete classes, which conceptually simplify system complexity^36,37^. If facies classes are defined based on properties (e.g., grain size distribution) with adequate spatial coverage and resolution, then they can be mapped over the domain of interest with quantified uncertainty. If the facies are also well-correlated to quantitative properties of interest required by numerical modeling (e.g. hydraulic properties, microbiologic characteristics) then it is possible to provide estimates of important properties that are more difficult or costly to measure. By investigating the spatial distribution of the facies and their properties, we can map the properties needed for numerical modeling of hyporheic exchange between groundwater and river water and associated biogeochemical transformations. Given the availability of sediment information either from direct measurements or inferred from hydrodynamic attributes, it is reasonable to define sediment-based facies for characterizing the HZ and mapping biogeochemical and microbial community properties across the HZ landscape in order to develop larger scale models of hyporheic zone processes.

To investigate these relationships in the hyporheic zone at the Hanford 300 Area along the Columbia River, grain size distribution, as a main determinant of bed permeability, was estimated for 21 freeze core samples taken just below the water line along 320 m of shoreline, and the relationships between geophysical and biochemical properties were determined. Network analysis was used to determine correspondence between microbial population structure, microbial activity, sediment texture and facies, and geochemistry. We hypothesized that: 1) sediments with increased hydraulic conductivity should have higher available carbon due to higher recharge rates and low residency times; 2) this, in turn, stimulates microbial activity resulting in higher biomass; 3) microbial community composition and function responds to hydrologic fluxes, with aerobic organisms and activities (such as respiration and nitrification) increasing with increased conductivity and anaerobic organisms and processes decreasing with decreased conductivity. Our results provide quantitative relationships between geology, geochemistry, and microbiology which can be applied to mapping biogeochemical distributions and modeling hyporheic flow and reactive transport processes. These relationships help understand the possible mechanisms of the geological control on biogeochemical processes, for example, by influencing surface area available for colonization, pore accessibility, and/or hydrologic recharge rate for oxygen availability.

## Materials and Methods

### Study Site

**Figure 1:**
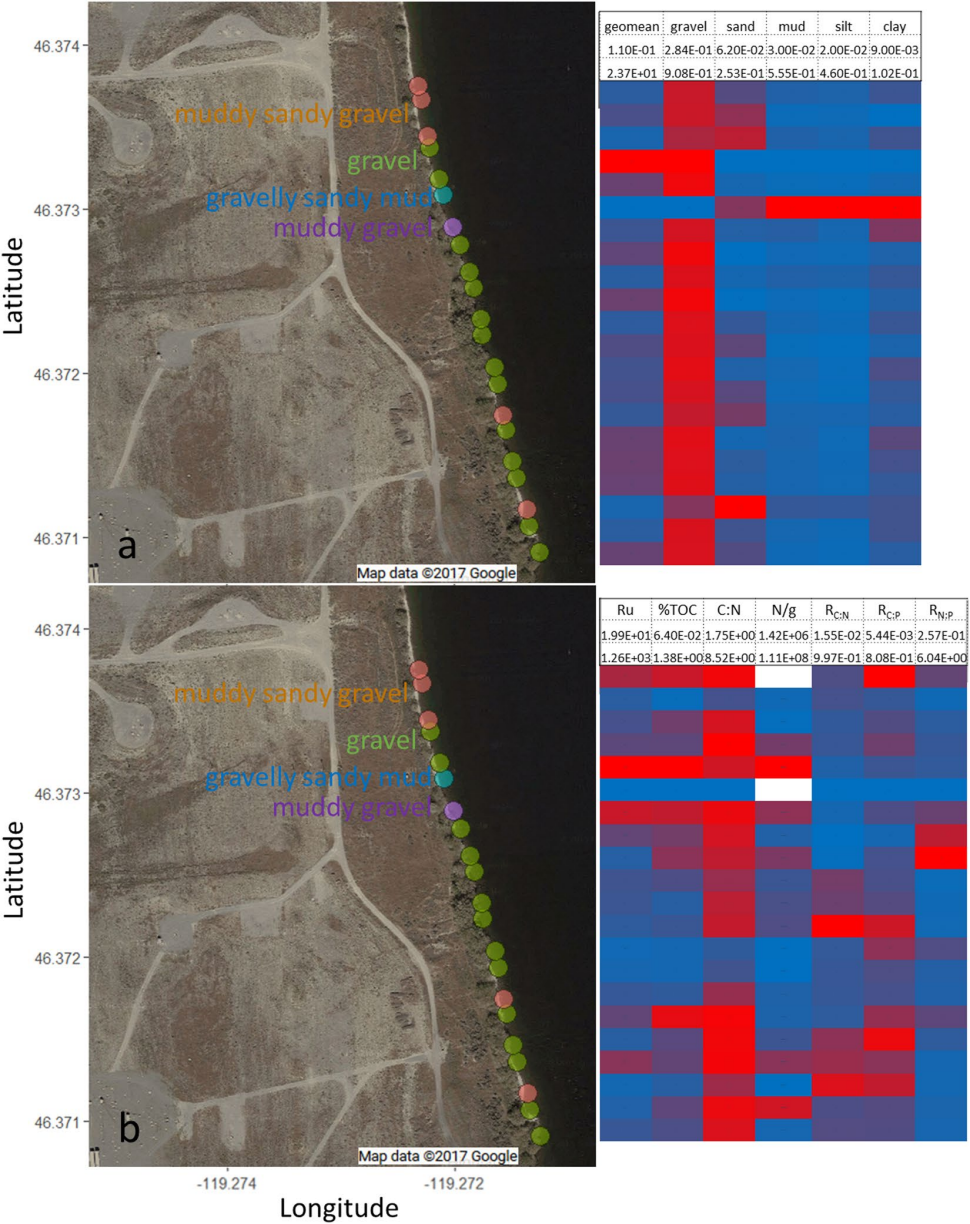
The spatial distributions of freeze core samples and their attributes: (**a**) geologic properties (geometric mean grain size, and percentages of gravel/sand/mud/silt/clay); (**b**) biogeochemical properties, See Table 2 for a description of biogeochemical parameters measured. The blue to red color scale correspond to measured parameter values from the lower to upper bounds, which are listed below the parameter name.

The study area is located in the Hanford 300 Area, with a spatial coverage of 320 m in the North-South direction, and 75 m in the East-West direction, along the Columbia River shoreline (see Fig. 1).

### Freeze coring

Cores were collected on the 9^th^, 10^th^ and 16^th^ of October 2014, using methods outlined in Moser *et al*.^38^ by driving a stainless steel tube (3.3 cm O.D., 2.4 cm I.D.) into the river bed and pouring in liquid nitrogen. The tube, together with the attached frozen sediment core surrounding the tube, were removed using a chain hoist and tripod. The average diameter of the cores was approximately 15 cm. Frozen sediment was detached with a mallet, wrapped in aluminum foil, and stored on dry ice. Three cores, ~1 m apart, were collected at each of 21 locations; two for chemical and biological analysis, and one for particle size distribution. Samples were stored at −80 °C.

### Freeze core processing and subsampling

Cores were thawed at room temperature for 1.5–3 hr and placed on a clean 2 mm sieve. Large rocks and pebbles were pared away using a sterile metal scoop to reveal uncontaminated surfaces. Sediment fractions (>~0.8 mm) were collected into a sterile stainless steel pan and mixed to homogenize. Two ~5 cm^3^ sediment samples for Deoxyribonucleic Acid (DNA) analysis were flash frozen in a dry ice/ethanol bath and stored at −80 °C. One ~5 cm^3^ sample for enzyme assays was stored at 4 °C. Four ~1 cm^3^ samples were collected into pre-weighed 20 ml amber vials (Thermoscientific 139–20 A/EP, Miami,OK.) for resazurin reduction incubations and stored at 4 °C. Remaining sediment was flash frozen and stored at −80 °C.

### Grain Size Distribution

Samples were transferred from −80 °C to a drying oven, dried for 4–5 days at 105 °C, weighed and sieved into phi size classes from 64 mm (−6 phi) to <0.062 mm (5 phi)^39–41^. The weight percentage for each size fraction was determined.

The <2 mm (0 phi) fractions were recombined and 10 g subsamples taken for laser diffraction analysis^42,43^. Samples were incubated overnight in 25 ml of 1% pyrophosphate with shaking. The sand fraction was determined by the dry weight percentage of material retained on a #230 sieve (0.062 mm). The unretained “slurry” was agitated then injected into the laser compartment for a triplicate measurement. The dried sand fraction was also evaluated in triplicate. Measurements were averaged, and the weight-by-size fractions of the original 10 g were calculated.

### Elemental analysis

Sediment samples were dried in a Blue M Stabil-Therm convection oven (Blue Island, IL) for 96 hrs at 45 °C, ground and sieved to 150 µm. Subsamples were taken (30–50 mg) and analyzed for total C, H, N, S and TOC using an Elementar vario EL Cube CHNS equipped with an autosampler (Elementar Analysensysteme GmbH, Hanua GE).

### Resazurin reduction assays

Replicate 1 cm^3^ samples (4) were supplemented with 6 ml of filtered (0.2 μm) river water in a sterile hood. One replicate was heat killed in a 90 °C water bath for 1 hr and cooled on ice to serve as a negative control. Assays were performed by adding 200 μL of 30 micromolar resazurin for a final concentration of ~1 μM, and incubating at 21 °C for 4 hrs with shaking (50 rpm). Reaction products were extracted by adding an equal volume of acetonitrile, vortexing, sonicating for 10 mins, and incubating for 1 hr as above. Sediment was allowed to settle, and supernatants were filtered, using 33 mm 0.2 μm syringe filters (PES, Millex by Millipore), into 12-mL amber vials (Thermoscientific) and stored at 4 °C until fluorescence measurement. Fluorescence emission maxima for resazurin (630 nm) and resorufin (585 nm) were measured on sample extracts using a Horiba Fluorolog 3 fluorimeter. For each sample, 2 mL of extract was added to 0.2 mL 100 mM HEPES (pH 8) in quartz cuvettes and fluorescence intensity quantified by comparison to resazurin and resorufin standards made up in ACN:H_2_O (1:1).

### pH

Samples were dried at 35 °C for 72 hrs and sieved to <1 mm. 2 ml of Milli-Q ultrapure water (EMD Millipore, Darmstadt, GE) was added to 2 g sediment. The sediment/water mixture was vortexed for 5 seconds then allowed to settle for 10 minutes. The pH of the resulting slurry was determined using a Denver Instruments Model 215 pH meter with a pH/ATC probe (Denver Instrument Company, Denver CO).

### Biological activity

Extracellular enzyme activity was determined using the method described by^44^, with optimizations for our material, environmental conditions (pH, temperature) and target enzymes.

Extracellular enzymes assayed include; β-glucosidase (200 µM 4-MUB-β-D-glucoside), N-acetyl glucosaminidase (200 µM 4-MUB-N-acetyl-β-D-glucosaminide), phosphatase (200 µM 4-MUB-phosphate), and aminopeptidase (200 µM L-Leucine-7-amino-4-methylcoumarin). Standards were made with 4-methylumbelliferome (MUB) and 7-Amino-4-methylcoumarin (AMC). All substrates and standards were purchased from Sigma Aldrich (St. Louis, MO).

For each sample, three, 0.25 g (wet) subsamples were weighed into 50 ml Corning Centrifuge tubes (Sigma Aldrich, St. Louis MO). 37.5 ml 50 mM MOPS buffer (pH 7.2) was added to each tube and vortexed for 1 minute. 200 µL aliquots of the suspension were transferred to a 96-well plate. In addition to the suspension, sample wells received 50 µL substrate, wells designated for quench standards received 50 µL substrate standards (10 µM, 5 µM, 2.5 µM, 1.25 µM, 0.625 µM, 0.313 µM, 0.156 µM, 0.078 µM, 0.0038 µM). Blank wells received 200 µL sample suspension and 50 µl buffer. Plates were incubated in the dark for 1.5 hr at ~21 °C. 10 µL aliquots of 1 M NaOH were added to each well to stop the reaction. Fluorescence was read with 365 nm excitation and 450 nm emission filters on a SpectrMax GeminiXS Microplate Spectrofluorometer (Molecular Devices, Sunnyvale CA). Activities were calculated on a per gram (dry weight) per hour basis. The measured properties include biomass and microbial activity parameters.

### Quantitative analysis of microbial abundance

Quantitative PCR (qPCR) was used to quantitate Bacterial 16 s rRNA gene copies in the genomic DNA samples. A general domain-level Bacterial assay (Nadkarni *et al*.^45^), was performed. Hydrolysis-probe assays and a double-stranded DNA standard was synthesized using gBlocks Gene Fragments by Integrated DNA Technologies (IDT; Coralville, Iowa (IDT). The standard was 924 bp in length, and consisted of a slightly modified portion of the 16 S rRNA gene of Escherichia coli^45^. Standard curves were generated across 6 orders of magnitude, and efficiency was 93%. Assays were performed in 384-well plates using a Life Technologies ViiA7 real-time PCR instrument at the DNA Services Facility at the University of Illinois at Chicago, using conditions described previously^45^. Final bacterial 16 S rRNA gene abundance was calculated from the linear regression of the standards, and copies per total extract was calculated by multiplying by the elution volume. Final normalization was to grams of dry sediment.

### Genomic DNA isolation

Subsamples were removed from −80 °C storage and thawed in an ice bucket for ~1hr. Excess water was removed by centrifugation for 1 min at 10,000 x g. Samples were split, and dry mass and moisture content was determined on one subsample by drying for 72 hrs at 65 °C. The moisture content was then used to calculate the dry mass of the other subsample. Genomic DNA was extracted using a MoBio PowerSoil kit (MoBio Laboratories, Inc., Carlsbad, CA). Extractions were done following manufacturer’s instructions with the addition of a 2 hr proteinase-K incubation to facilitate cell lysis. Briefly, 2 µl of 20 mg/ml proteinase-K solution (Applied Biosystems) and 60 µl of C1 solution was added to each bead tube, vortexed for 5 sec and then placed in a 60 °C water bath for 1hr.

### Community structure analysis

In phylogeny, an operational taxonomic unit (OTU) is an operational definition of a species or group of species often used when only DNA sequence data is available. It is the most commonly used microbial diversity unit. In this study, OTUs were derived from amplicon analysis of the V4 region of the 16 S ribosomal RNA gene (*rrnA*). Amplification from genomic DNA isolated from sediment samples was performed using the Earth Microbiome Project barcoded primer set 515 F/806 R (http://press.igsb.anl.gov/earthmicrobiome/emp-standard-protocols/16s/)^46^ with the exception that the twelve-base barcode sequence was included in the forward primer. Amplicons were sequenced on an Illumina MiSeq using the 300 cycle MiSeq Reagent Kit v2 (http://www.illumina.com/) according to manufacturer’s instructions.

Resulting sequences were processed with QIIME^47^. The split_libraries_fastq.py was used to de-multiplex the fastq-formatted sequences, and sequences were screened for a Phred quality score of 20. Trimmed sequences were analyzed using mothur v 1.34.0^48^. Sequences were aligned against the SILVA v 123 seed alignment (Silva.seed_v123.tgz) provided on the mothur website (http://mothur.org/wiki/Silva_reference_files). The alignment was trimmed to columns 13,862–23,444 with a maximum allowed homopolymer run length of 8. Sequences were pre-clustered with an allowance of 2 mismatches and checked for chimeric sequences using uchime^49^. This process resulted in 367,620 total sequences and 102,351 unique sequences. Sequences were classified against the SILVA v123 seed reference set using the default Wang method^50^. A distance matrix was built using the default onegap option and end gap penalties (countends = T), and the sequences were clustered using the default average neighbor algorithm. OTUs were defined at 97% identity. Diversity and sampling calculations were performed on data sets down-sampled to the size of the smallest set (N = 11,934).

### Network analysis

OTUs comprising > 0.1% total abundance of any of the samples (620 OTUs) were used in network analysis with the biogeochemical (BGC) data. The absolute abundance of organisms was estimated by multiplying the relative abundance values calculated from the amplicon data by the qPCR-derived *rrnA* copies/g dry sediment values. Biogeochemical data was log_e_-transformed. qPCR is widely used for DNA or ribonucleic acid (RNA) quantification in molecular biology with amplification of nucleic acid using a polymerase chain reaction (PCR)^51^. Network analysis was done using the WGCNA package^52^. OTU abundances were correlated using the ‘cor’ function and ‘spearman’ method. Dissimilarity was calculated (1 – abs(x)), and data was clustered using the ‘hclust’ function and the ‘average’ method. In biogeochemistry, the clusters of community composition data by network analysis, based on Spearman’s rank correlation, are named modules. Here, modules were defined using the ‘cutreeDynamic’ function with the options deepsplit = 2, pamRespectsDendro = F and minClusterSize = 10. The module eigengene of a given module is defined as the first principal component of the standardized gene expression profiles. Here, module eigengenes were extracted and correlated with the transformed BGC data, as described above. Correlations between OTUs and BGC data were also calculated as described. Node pairs for which the Spearman rho value magnitude was ≥ 0.6 and having a corresponding P-value ≤ 0.05 were exported to Cytoscape^53^ for visualization.

### Statistical analysis

Clustering of samples based on grain size distribution is critical as it provides the basis for defining facies and grouping components of the complex biogeochemical system into a manageable set of discrete classes. Geological classification was done following Folk’s classification system with the descriptive terminology modified from and the Udden-Wentworth grade scale^39,40^.

Alternative clustering approaches based on statistical algorithms were also explored, including Principal Component Analysis (PCA), hierarchical and Expectation-Maximization (EM) clustering. PCA was used for identifying similarities in the multivariate set of variables including physical, geochemical, and microbial community attributes. PCA reveals the internal structure of the data in a way that best explains the variance in the data. By using only the first few principal components, the dimensionality of the transformed data can be reduced. The built-in R function prcomp was used for PCA and the package ‘factoextra’ was used for visualization. The Expectation-Maximization (EM) algorithm is a general approach for approximating maximum likelihood estimations in a multivariate data set^54^. It provides objective clustering solutions and also offers uncertainty measures of the resulting classification^55,56^.

Multivariate Regression and ANalysis Of VAriance (ANOVA) approaches have been widely used in various disciplines to evaluate relationships between dependent variables and multiple explanatory variables, discrete or continuous^57–64^. Multiple regression can be displayed by plotting the dependent variable against the raw values of the independent variable. In practice, the procedure and resulting multiple regression coefficients may be misleading because the value of the partial slope may change in magnitude and even in sign relative to the slope obtained in simple least-squares regression. The issue can be even worse when the explanatory variables are dependent on or constrained by each other. One possible solution is to evaluate partial relationships with partial regression. Partial regression is useful for visualizing the true scatter of points around the partial regression line and identifying influential observations and non-linear patterns more efficiently than using plots of residuals vs fitted values. Partial regression analysis can be done by evaluating residuals of the dependent variable on the remaining explanatory variables vs residuals of the target explanatory variable on the remaining explanatory variables^65^.

## Results

### Associations of geological facies and biogeochemical properties

**Figure 2:**
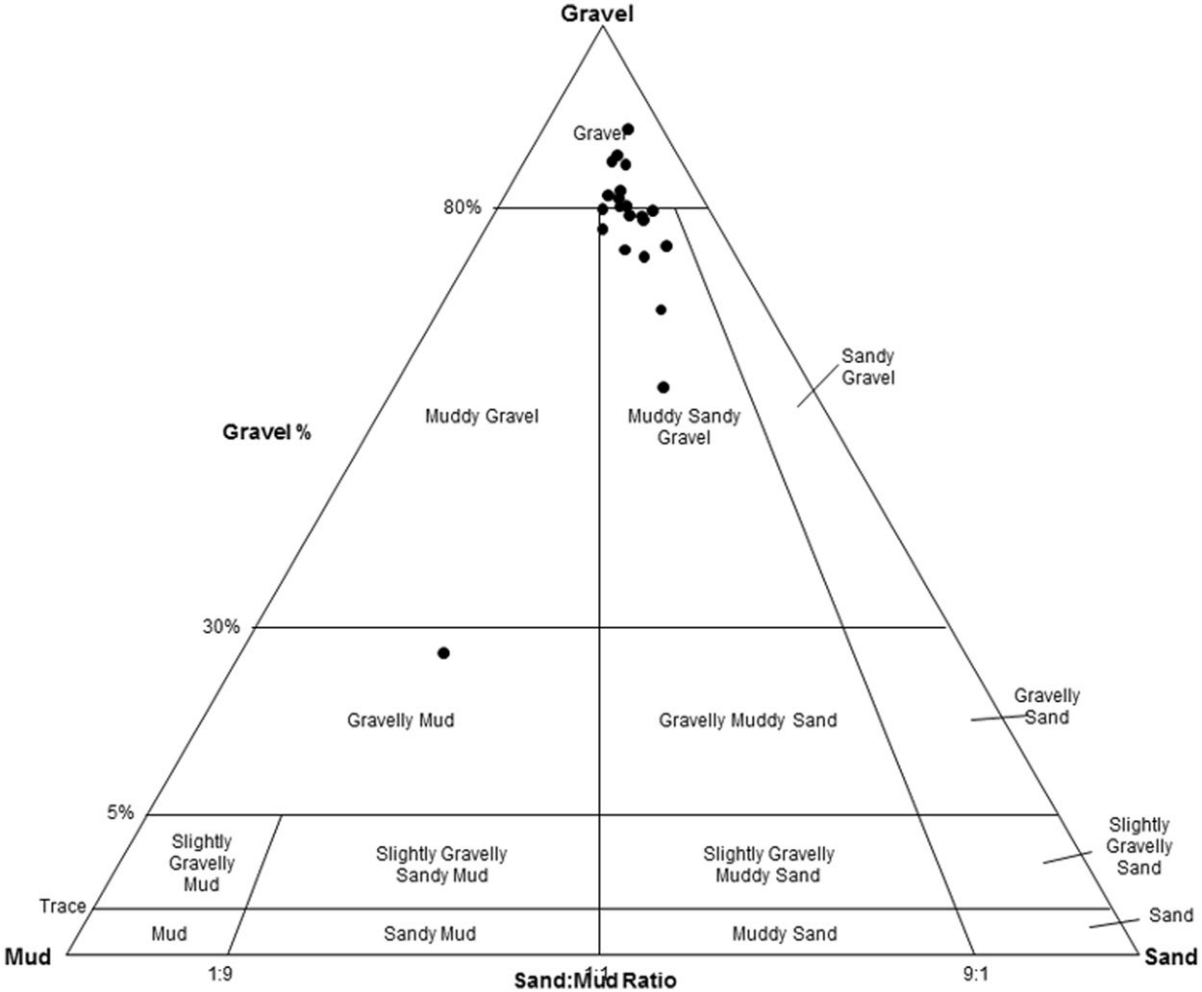
A plot of the grain size distributions of the samples (gravel, sand, mud) according to Folk’s classification system.

The relationships between geological attributes (grain size distribution [gsd] and gsd-based facies) and biogeochemical properties were evaluated. The freeze core sediment samples were generally dominated by gravel, with geometric average grain size ranging from 0.11 to 23.7mm. According to Folk’s classification system, the samples can be classified into four different classes/facies: gravel (G), muddy gravel (MG), muddy sandy gravel (MSG), and gravelly mud (GM), each containing 14, 1, 5, and 1 sample(s) respectively, as shown in Fig. 2. Folk’s classification (FC) is based on the relative ratios of gravel, sand, and mud. The grain sizes also exhibit different sorting: by adding sand, silt, and clay content, such that the samples can be further classified as very coarse gravel (11), coarse gravel (3), very coarse silty sandy very coarse gravel (1), coarse silty sandy very coarse gravel (3), muddy very coarse gravel (1), very coarse gravelly medium silt (1), and medium silty sandy coarse gravel (1), with the number in each parenthesis indicating the fractional mass of material in the class, as summarized in Table 1.

**Table 1:** Major geological properties from the core sample measurements. GRAVEL, SAND, MUD, SILT, and CLAY denote decimal fractions of each grain size. FC is the classification in Folk’s system^111,112^.

Sample ID	GRAVEL	SAND	MUD	SILT	CLAY	Grain.arith.mean.mm	Grain.geo.mean.mm	FC	Finer class
FC.01	0.813	0.124	0.063	0.04	0.024	32.171	9.694	Gravel	Very Coarse Gravel
FC.02	0.814	0.11	0.076	0.044	0.033	11.023	4.112	Gravel	Coarse Gravel
FC.03	0.608	0.253	0.14	0.111	0.033	21.559	2.552	Muddy Sandy Gravel	Very Coarse Silty Sandy Very Coarse Gravel
FC.04	0.832	0.09	0.078	0.05	0.028	30.938	9.776	Gravel	Very Coarse Gravel
FC.05	0.823	0.096	0.081	0.049	0.034	34.707	10.114	Gravel	Very Coarse Gravel
FC.06	0.835	0.078	0.087	0.046	0.042	34.361	9.896	Gravel	Very Coarse Gravel
FC.07	0.765	0.151	0.083	0.061	0.024	17.56	5.168	Muddy Sandy Gravel	Coarse Silty Sandy Very Coarse Gravel
FC.08	0.809	0.129	0.062	0.035	0.028	18.202	6.706	Gravel	Very Coarse Gravel
FC.09	0.841	0.087	0.072	0.036	0.037	22.021	6.303	Gravel	Very Coarse Gravel
FC.10	0.819	0.133	0.048	0.029	0.019	24.582	7.499	Gravel	Very Coarse Gravel
FC.11	0.824	0.102	0.074	0.044	0.03	10.93	4.401	Gravel	Coarse Gravel
FC.12	0.88	0.064	0.056	0.034	0.022	24.861	10.078	Gravel	Very Coarse Gravel
FC.13	0.821	0.078	0.101	0.073	0.029	11.728	3.946	Gravel	Coarse Gravel
FC.14	0.873	0.062	0.065	0.046	0.02	20.814	9.041	Gravel	Very Coarse Gravel
FC.15	0.799	0.089	0.112	0.058	0.055	23.81	5.744	Muddy Gravel	Muddy Very Coarse Gravel
FC.16	0.284	0.161	0.555	0.46	0.102	4.874	0.11	Gravelly Mud	Very Coarse Gravelly Medium Silt
FC.17	0.869	0.079	0.052	0.036	0.016	20.741	9.952	Gravel	Very Coarse Gravel
FC.18	0.908	0.062	0.03	0.02	0.01	46.069	23.732	Gravel	Very Coarse Gravel
FC.19	0.701	0.203	0.096	0.065	0.032	8.443	2.539	Muddy Sandy Gravel	Medium Silty Sandy Coarse Gravel
FC.20	0.778	0.17	0.053	0.045	0.009	19.294	6.994	Muddy Sandy Gravel	Coarse Silty Sandy Very Coarse Gravel
FC.21	0.774	0.126	0.101	0.071	0.031	15.382	4.236	Muddy Sandy Gravel	Coarse Silty Sandy Very Coarse Gravel
Min	0.284	0.062	0.03	0.02	0.009	4.874	0.11	—	—
Max	0.908	0.253	0.555	0.46	0.102	46.069	23.732	—	—

**Figure 3:**
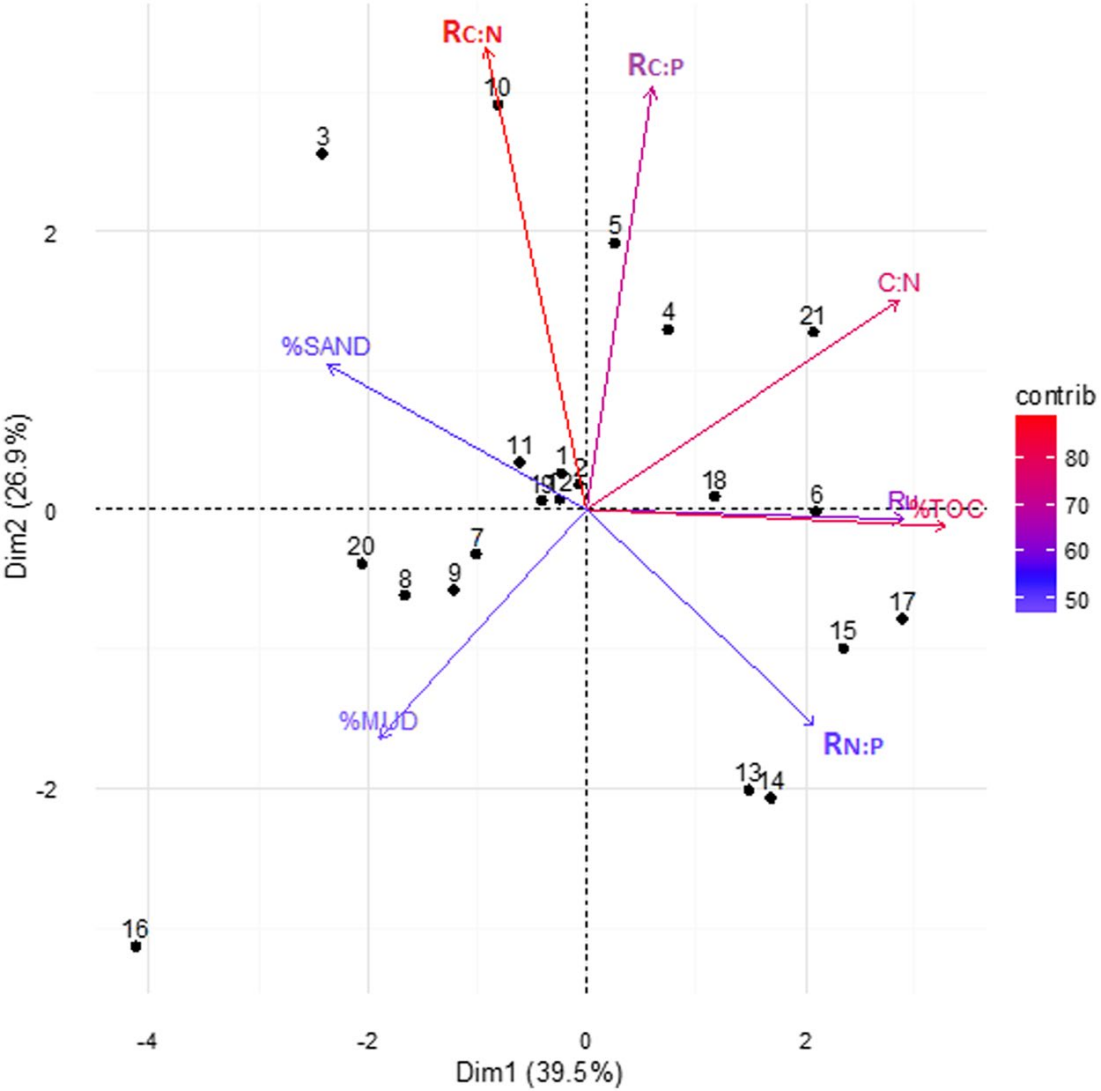
Principal component analysis: the projections of selected environmental variables on the first two principal components, where the colors and distances to the origin show the contributions of individuals (in percentage) to the first two principal components.

An alternative clustering approach to Folk’s classification is statistical EM clustering of the full range grain size distributions. Compared to the FC facies, the number of samples is more evenly dispersed among EM facies. There are different ways to quantitatively describe the grain size features, including the raw grain size percentages (e.g., %gravel, %sand, %mud), the classes based on the different attributes of gsd, and geometric and arithmetic averages of the grain sizes. These geological properties are all listed in Table 1, and any subset of these descriptors can be included for evaluating their control on biogeochemical variations. However, if the complete set of these physical attributes are used as explanatory variables, their relative contributions cannot be reliably resolved because of the closure problem (i.e., the constant sum of the grain size fractions). This occurs because the fractions of the different grain size classes sum to one, inducing spurious negative correlations between the grain size classes. Therefore, a parameter screening was done by performing paired individual regression between the physical attributes and all environmental variables, and the two most influential parameters were identified as %sand and %mud, which are nearly orthogonal to one another (Fig. 3).

The complete environmental variables and measurements are listed in Supplemental Table S1. Dependent variables selected for further analysis, as shown in Table 2, include those with strong associations with either C/N/P concentration, or C/N/P-mining enzyme/activities. Multivariate relationships among measured variables are visualized across the first two PCA axes (the first two PC’s explained 66.5% of overall variability) in Fig. 3, which shows that samples don’t cluster and most variables don’t co-vary. Furthermore, it shows that C:N ratio has a negative correlation with %mud, and that R_N:P_ (the ratio of *N*-acetyl-glucosaminidase and aminopeptidase to phosphatase activity) has a negative relationship with %sand; while Ru and %TOC are positively correlated.

**Table 2:** Major biogeochemical properties from the core sample measurements. Variable names are defined below.

Sample ID	Ru^a^	%TOC^b^	C:N^c^	N/g^d^	R_C:N_^e^	R_C:P_^f^	R_N:P_^g^
FC.01	292.205	0.461	7.5417	11031250.8	0.336333	0.265602	0.789701
FC.02	185.291	0.550	7.9727	90418953.57	0.313448	0.273874	0.873747
FC.03	116.924	0.274	5.9616	2086784.735	0.86347	0.613372	0.710357
FC.04	683.271	0.573	8.2224	60993878.05	0.606093	0.440953	0.727534
FC.05	216.037	0.528	8.1755	28005721.73	0.560413	0.750159	1.338582
FC.06	494.495	1.261	8.2987	14159947.05	0.199162	0.473203	2.375969
FC.07	285.960	0.313	5.7482	15127793.05	0.230608	0.229859	0.996751
FC.08	134.847	0.172	3.4881	1416837.843	0.232078	0.254128	1.095012
FC.09	103.756	0.173	3.0438	2278497.535	0.216065	0.451179	2.088162
FC.10	247.441	0.367	6.9649	36104904.28	0.996591	0.648443	0.650661
FC.11	310.980	0.271	6.7094	33486500.49	0.438069	0.274565	0.626761
FC.12	334.371	0.465	5.7674	27059703.57	0.46208	0.236422	0.511648
FC.13	204.871	0.785	6.8386	54922288.74	0.037236	0.225059	6.044083
FC.14	504.040	0.663	7.3737	16641042.27	0.015526	0.070871	4.564779
FC.15	995.172	1.062	7.9962	62913772.75	0.09452	0.250223	2.6473
FC.16	19.915	0.064	1.7546	NA	0.021143	0.005438	0.257217
FC.17	1259.379	1.381	7.2506	110810426.6	0.140351	0.195613	1.393737
FC.18	473.577	0.552	8.5225	51329850.97	0.234754	0.352666	1.502278
FC.19	361.960	0.646	7.4221	3907789.383	0.211445	0.266041	1.258202
FC.20	224.686	0.109	3.5707	8510738.657	0.284515	0.181567	0.638162
FC.21	841.796	1.099	8.217	NA	0.319868	0.808347	2.527127
Min	19.915	0.064	1.7546	1416837.843	0.015526	0.005438	0.257217
Max	1259.379	1.381	8.5225	110810426.6	0.996591	0.808347	6.044083

^**a**^Resazurin-resorufin assay per dry gram of sediment, a proxy for aerobic respiration rate. ^**b**^Total organic carbon content. ^**c**^Carbon:nitrogen ratio. ^**d**^Copies of *rrnA* detected per gram (dry weight) of sediment as determined by qPCR; a measure of bacterial and archaeal total abundance. ^**e**^Ratio of β-glucosidase activity to N-acetyl-glucosaminidase + aminopeptidase activities; a measure of whether an ecological system is controlled by energy flow or N limitation. ^**f**^Ratio of β-glucosidase activity to phosphatase activity; as above, but for P limitation. ^**g**^Ratio of N-acetyl-glucosaminidase + aminopeptidase activities to phosphatase activity; as above, but comparing the relative strength of control by N versus P.

PCA shows that %mud and %sand are essentially orthogonal, but still they are slightly correlated with each other due to the closure problem. Therefore, partial regression analysis was performed to study the ‘true’ relationships between the predictors (i.e., %sand, %mud) and dependent variables to avoid misleading regression coefficients and relationships.

**Figure 4:**
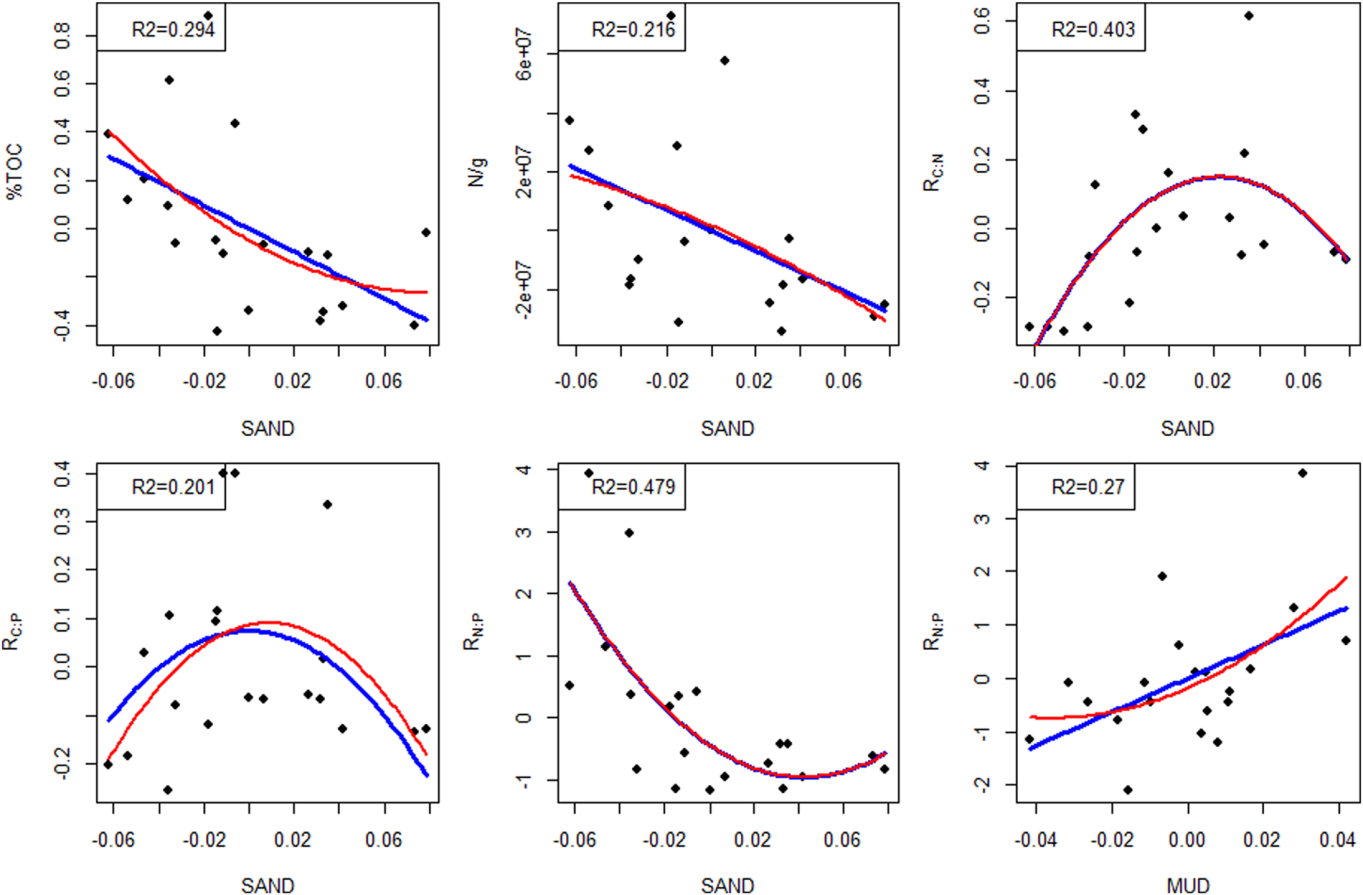
The partial regressions of the selected environmental variables with respect to sand and mud content. Note that in partial regression analysis, the dependent variables are fitted using residuals of the target explanatory variable on the remaining explanatory variables. The figure only shows the relationships with R^2^ > 0.2, and the dependent variables shown are unitless ratios or percentages (see definitions in Table 2).

In order to understand whether the %sand and %mud have nonlinear relationships with biogeochemical attributes, the partial regression starts with a quadratic model and then model selection techniques are used. Specifically, the initial partial regression models include intercept, linear, and second order terms. Then the Akaike Information Criterion (AIC) was used for backward removal of unnecessary terms^66^. The final model represents the most parsimonious model of the true relationship between the dependent variable and the predictors (see Fig. 4). In Fig. 4, the initial models are in red and the final models are in blue. Only one line is shown if the initial quadratic model cannot be reduced. The results show that most biogeochemical attributes have good linear correlations with %sand and several of them also correlate well with %mud. Exceptions to this include microbial activity parameters such as R_N:P_ and R_C:N_ (the ratio of beta-glucosidase to *N*-acetyl-glucosaminidase and aminopeptidase activity), which have strong nonlinear relationships with %sand (the R^2^ for fitting the variations of the two parameters are 0.479 and 0.403 respectively). As R^2^ represents the amount of uncertainty that can be explained by a relationship, such a percentage reduction in uncertainty is very useful when constructing reactive transport model inputs compared to mapping without grain size information and assuming a homogeneous spatial distribution of BGC properties.

### Relation of microbial taxa (or sets of taxa) to facies

#### Microbial community composition

Amplicon analysis of the 16 S rRNA gene was performed on genomic DNA isolated from the 21 freeze core samples to examine how the native microbial community structure varies across the sampled facies. Good’s coverage estimates suggest the sequencing depth sampled between 84 and 92% of the total community (Table 3). Species richness and diversity were similar across all samples, with Chao1 richness estimates ranging from 5143 to 12679, and the inverse Simpson index ranging from 44 to 306. Membership was similar across samples at the Phylum level with 11–19% Acidobacteria, 9–22% Proteobacteria, 7–18% Thaumarchaea, 5–19% Nitrospirae, 3–17% Actinobacteria, 5–13% Chloroflexi, 4–12% Planctomycetes, 1–8% Verrucomicrobia, 1–5% Latescibacteria and 5–8% unclassified Bacteria (Figure S2A). A distance decay analysis of β diversity (Bray-Curtis) indicated a negligible effect of spatial separation on community composition (Figure S3, R^2^ = 0.055).

**Table 3:** Microbial diversity of freeze core communities.

Sample	Sequences collected^*a*^	Good’s coverage	OTUs observed	Chao1 richness estimate	Inverse Simpson index
FC01	28706	0.89	5416	11574	93.998
FC02	21041	0.86	4720	11850	159.445
FC03	18690	0. 90	3365	6987	165.24
FC04	16789	0.88	3182	7878	110.661
FC05	22433	0.88	4435	10144	174.794
FC06	18806	0.89	3086	8283	56.825
FC07	17349	0.87	3432	8741	72.194
FC08	20345	0.89	3742	7967	167.963
FC09	—	—	—	—	—
FC10	39821	0.92	5882	12679	87.451
FC11	21444	0.89	4053	9167	177.713
FC12	—	—	—	—	—
FC13	19142	0.91	3019	6379	123.157
FC14	15884	0.91	2479	5413	65.255
FC15	15555	0.88	2833	7012	79.998
FC16	—	—	—	—	—
FC17	23738	0.86	5111	12201	205.535
FC18	11934	0.88	2463	5143	146.111
FC19	25079	0.91	3484	8821	48.061
FC20	15361	0.84	4123	9121	305.737
FC21	15497	0.90	2632	5345	43.983
Min	11934	0.84	2463	5143	43.983
Max	39821	0.92	5882	12679	305.737

*a* after QA/QC.

**Figure 5:**
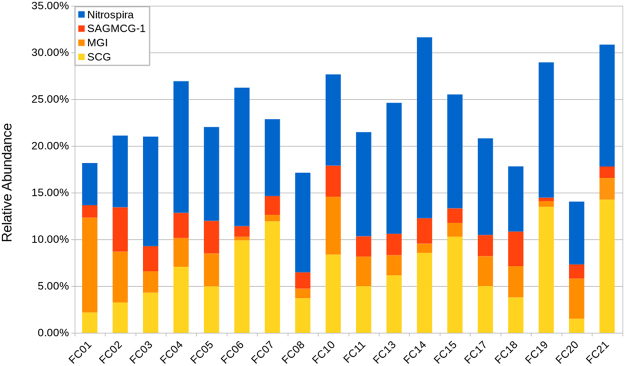
Distribution of Thaumarchaea and Nitrospira OTUs. Relative abundance based on *rrnA* amplicon sequence analysis. OTU clustering was performed with a 97% similarity cutoff using mothur. Taxonomic identification used Silva v123 as a reference.

The Thaumarchaeota and Nitrospirae appear to be less diverse than other clades (Figure S2B), with 5 OTUs comprising 85% of summed Thaumarchaeal abundance and 5 OTUs comprising 70% of the Nitrospira. For comparison, the top 5 Acidobacterial OTUs comprise only 27% of the Acidobacteria; in contrast, the top 5 Proteobacterial OTUs comprise only 16% of the Proteobacteria. A Class-level examination of the distribution of Thaumaracheal OTUs showed variation in composition across sites, with the most upstream sites (FC01 and FC02) containing more OTUs of the Marine Group I (MG-I), while most other samples contain more Soil Crenarchaeal Group (SCG) OTUs (Fig. 5). Described Thaumarchaeota are ammonia oxidizers, and Nitrospira are nitrite oxidizers, thus together they complete the nitrification pathway from ammonia to nitrate. Despite this potential for cooperative metabolism, there was not a strong relationship between overall Thaumarchaeal abundance and Nitrospiral abundance (Figure S4, R^2^ = 0.065). Analysis at the Class level, though, revealed a positive correlation between SCG abundance and Nitrospira (R^2^ = 0.309), and a negative correlation between MG-I abundance and Nitrospira (R^2^ = 0.207) (Figure S4).

**Figure 6:**
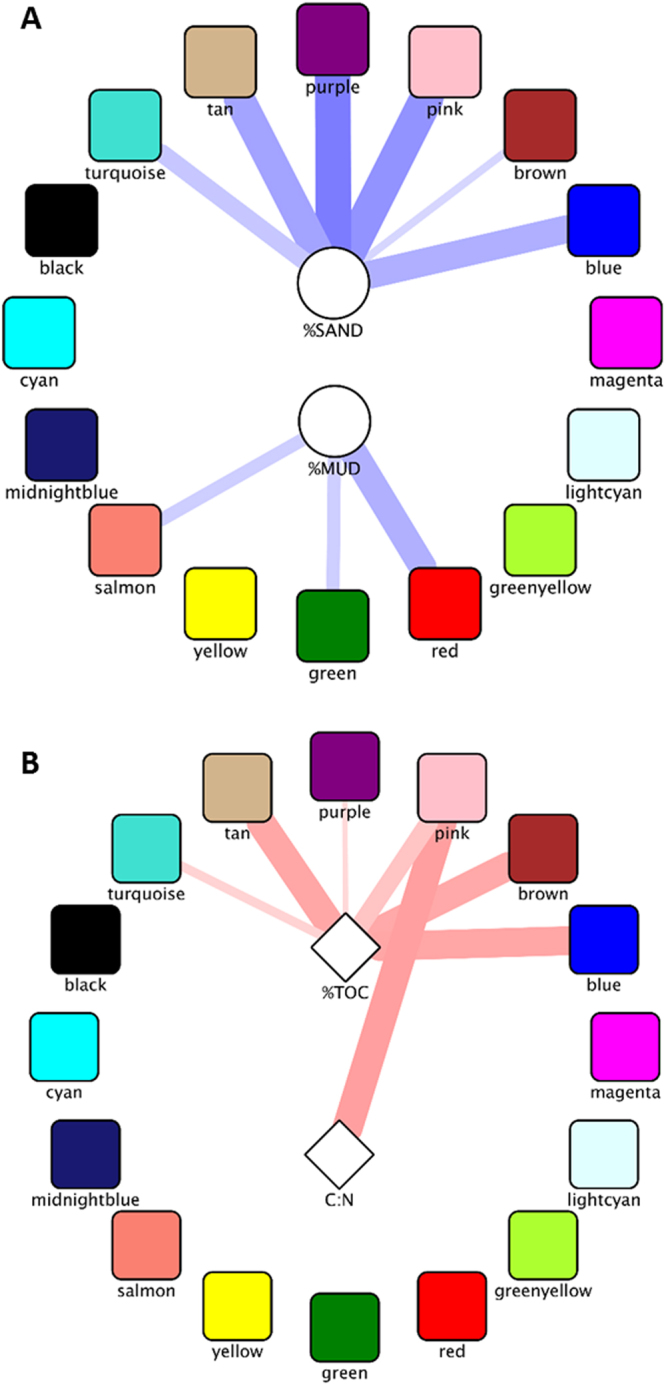
Correlations between biogeochemical properties and organismal modules. Modules were defined by network analysis on community composition data based on Spearman’s rank correlation, clustering OTUs that co-vary across samples. Eigengenes of the modules were then correlated against biogeochemical properties. (**A**) Geophysical properties. (**B**) Chemical properties. Red edges indicate a positive Spearman rank correlation, while blue edges denote a negative correlation. Edge hue intensity indicates magnitude of the Spearman rho value. Edge width indicates p-value, with p = 0.05 for narrowest line and p ≤ 0.01 for the widest lines.

To examine the relationship between biogeochemical parameters and community composition, network analysis based on Spearman rank abundance was performed on the most abundant OTUs in the data set (comprising at least 0.1% of the community in at least one sample). First, the OTUs which were observed to co-vary in abundance across the sample set, and thus are assumed to either respond to similar stimuli or exhibit cooperative growth, were grouped, yielding sixteen ‘modules’ which were randomly assigned color designations. Co-variance between two organisms can manifest either positively, with both increasing in abundance together and decreasing together, or negatively, with opposing increases and decreases. Relationships between OTUs within modules were almost all positive (data not shown). Because the organisms in the modules respond in concert, they can be considered as a single ecological unit to simplify analysis. Modules were correlated with measured environmental parameters (described above) classified into three types: the physical parameters %sand and %mud, as indicators of hydraulic conductivity^67–69^ (Fig. 6A), the chemical parameters %TOC and C:N, as measures of carbon and nitrogen availability (Fig. 6B), and the biological parameters Ru, R_C:N_, R_C:P_ and R_N:P_. No significant correlation between the modules and the biological parameters was observed.

We observed strong correlations between the turquoise, tan, purple, pink, brown, and blue modules and %sand and between the red, green and salmon modules and %mud. No modules correlated with both, and, surprisingly, all significant correlations were negative. The same modules that correlated with %sand also had significant positive relationships with %TOC, in agreement with relationships observed in Figs 3 and 4, while the pink module also positively correlated with C:N ratio.

**Figure 7:**
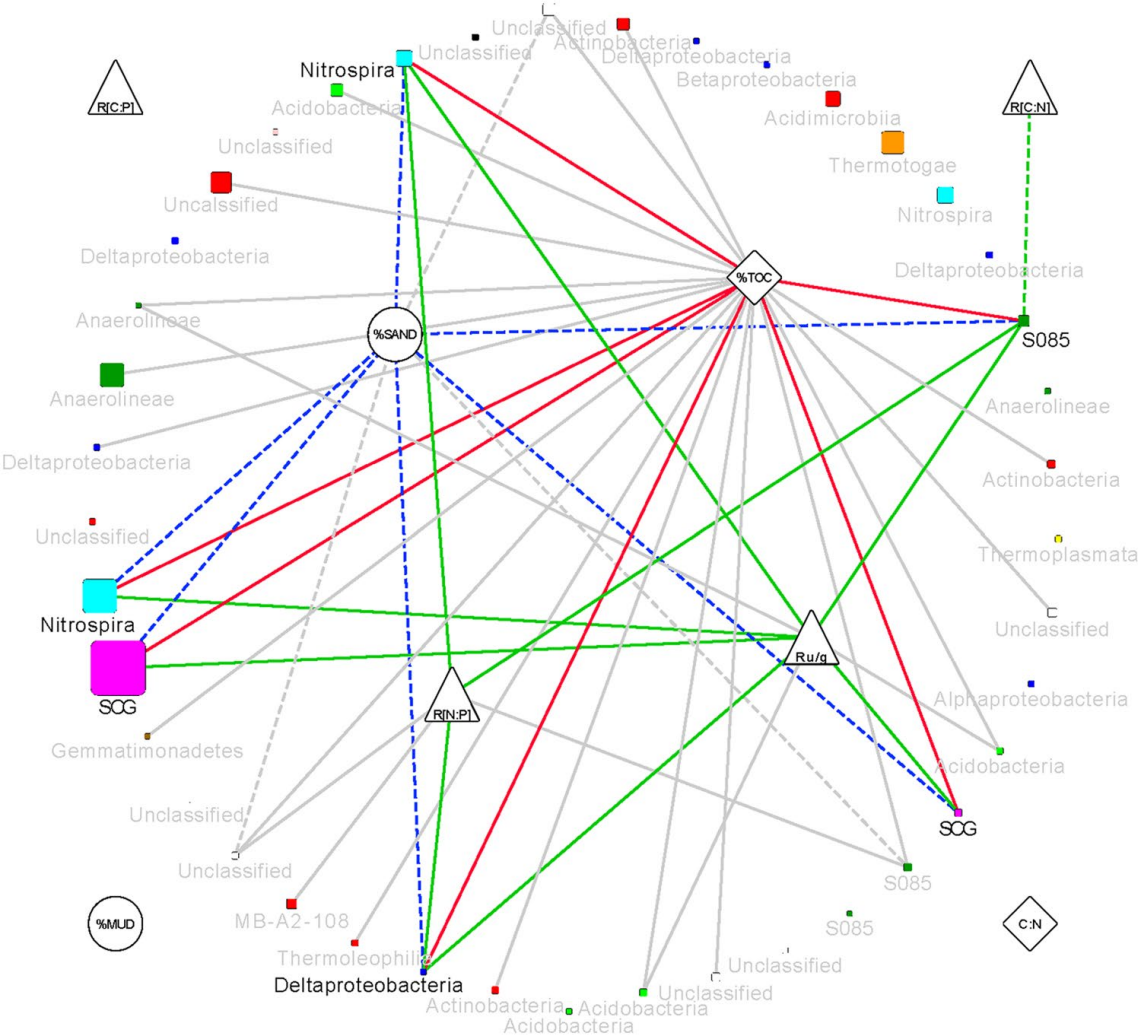
Network plot showing correlations of OTU members (rounded squares) of the brown module to the geophysical (circles), chemical (diamonds) and biological (triangles) parameters measured. OTU node size indicates estimated absolute abundance averaged across all samples, and color indicates Phylum association: magenta – Thaumarchaeota, cyan – Nitrospirae, light green – Acidobacteria, red – Actinobacteria, dark green – Chloroflexi, yellow – Euryarchaeota, cyan – Planctomycetes, blue – Proteobacteria, orange – Thermotogae, brown – Gemmatimonadetes, pink – JL-ETNP-Z39, black – Latescibacteria, and gray – unclassified. OTU nodes are labeled with their Class designation. Dashed edges denote negative relationships; solid edges denote positive relationships. Nodes with significant relationships to carbon (%TOC), texture (%SAND), and oxygen consumption (Ru/g) and their associated edges are highlighted. Edge colors denote connections to geophysical (blue), chemical (red) and biological (green) properties.

The brown module contained 29 OTUs, including the most overall abundant OTU within the data set, a SCG OTU, and three abundant Nitrospira OTUs (Table S2). These dominant taxa, save one of the Nitrospira OTUs, have strong individual correlations with %sand, and %TOC, likely driving the overall relationship between the brown module and these properties (Fig. 7). Over half (23) of the member OTUs had a significant positive correlation with %TOC, including presumed heterotrophs, such as the Acidobacteria and Actinobacteria, and autotrophs (the dominant SCG, two of the abundant Nitrospirae). A subset of these organisms was also associated with resazurin reduction, including not only two Acidobacteria, a Deltaproteobacterium (Desulfurellaceae), and two Chloroflexi (one of which was an Anaerolineae), but also the Thaumarchaeota and Nitrospirae, suggesting nitrification is a significant component of the aerobic respiration measured by resazurin reduction.

## Discussion

**Figure 8:**
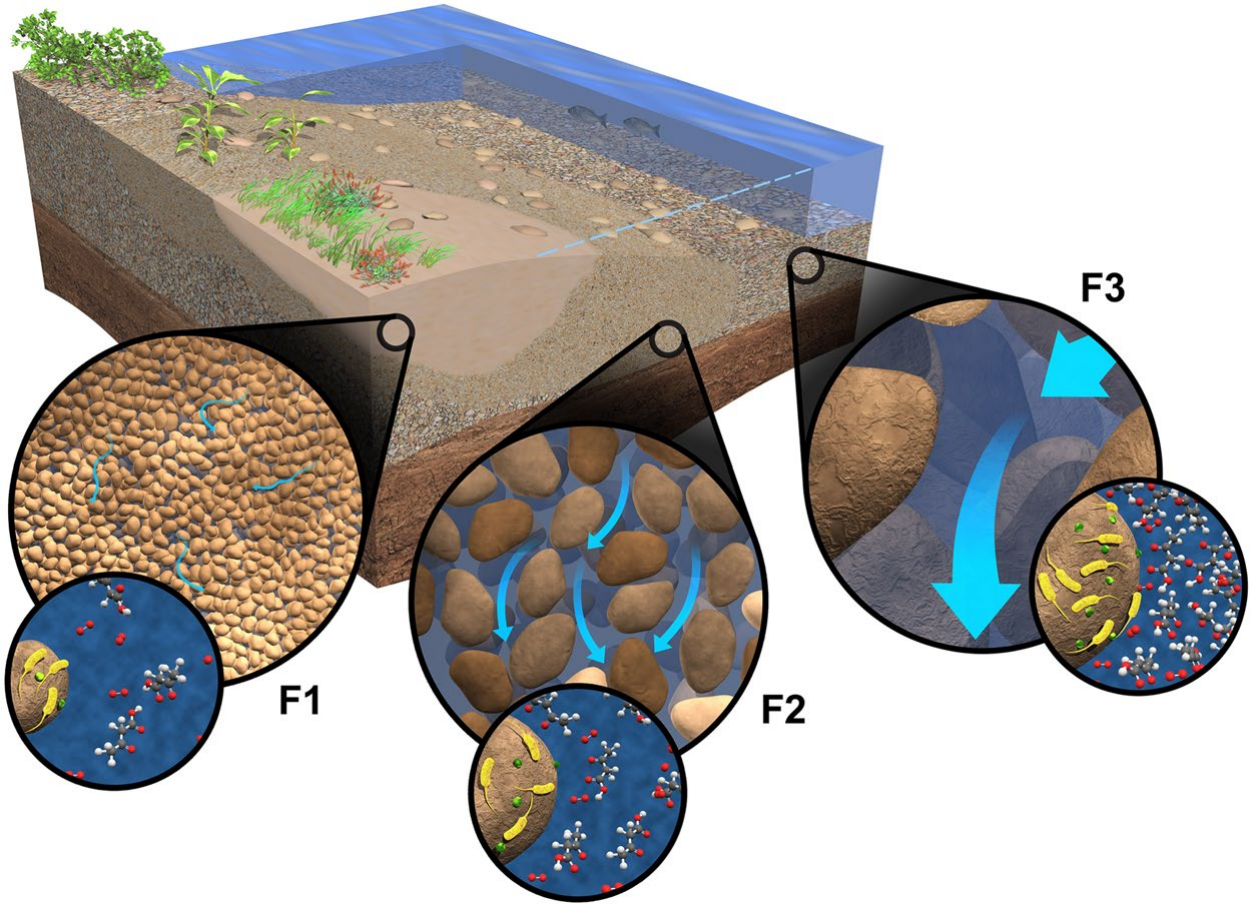
Conceptual model for relationships among textural, hydrological, and biogeochemical properties (e.g., flow rate, hydraulic conductivity, biomass, oxygen, organic compound). Higher flow rates through HZ facies with coarser particle size (and thus higher permeability) result in greater input of O_2_ and organic carbon in those facies, which stimulates higher microbial biomass and increased heterotrophic activity.

Geophysical properties of sediments modulate hydrologic flow that in turn influences the distribution and dynamics of microbial populations and biogeochemical attributes in the HZ^1,9^. The relationships between sediment size and permeability have been explored extensively in literature^28,70,71^, which generally indicate higher permeability for coarser materials. This relationship is routinely used to estimate permeability from grain size distributions for modeling exchange flows in the HZ^29,68,72^. In the Columbia River HZ, we propose a model in which areas of higher hyporheic exchange associated with larger average particle sizes (e.g., at least 10 s of mm in diameter) have higher sustained O_2_ and organic carbon concentrations due to higher connectivity with surface waters (see Fig. 8). This nutrient-rich environment promotes higher microbial biomass and increased heterotrophic respiration. Heterotrophic metabolism can release NH_4_, depending upon the C:N ratio of the organic matter, subsequently stimulating nitrification^73,74^. Therefore, in our model, aerobic metabolic rates are considered to be higher in facies with lower amounts of fine-grained materials. Increased fine particle content (i.e., %mud) in the HZ increases surface area available for colonization and can promote higher microbial abundances^75^, but, fine-grained sediments also have very low pore connectivity, limiting microbial activity due to slow recharge rates for O_2_ and nutrients; the interplay of these mechanisms make the relationship between particle size and microbial activity site-specific^76^.

The correspondence between sand and mud contents and various biogeochemical attributes (e.g., %TOC, R_C:N_, R_N:P_) suggest that grain size-based FC facies also have good correspondence to BGC characteristics, and therefore an efficient way to infer BGC properties is to utilize the statistical distributions of BGC properties with respect to grain size-based facies. The information is particularly needed when modeling is extended to larger spatial scales where both hydrogeological and biogeochemical properties are difficult to discern with adequate spatial coverage and resolution. Sediment grain size, however, is more easily obtained or inferred (e.g., via sieving or imaging). The fact that %sand can explain more of the variability of biogeochemical attributes than %mud could be site-specific to the study area and related to the structure (e.g., compactness and connectivity) of the sediment sampled in the current study. Comparison of classifications showed that Folk’s classification was more consistent than hierarchical or EM clustering, as the Folk’s groupings do not change when more samples were added for facies definition. This is a desirable feature for characterizing the HZ at various locations and larger scales. Moreover, ANOVA were performed by considering the Folk classes (G, MG, MSG, GM) as factors, and similarly for the other two classification schemes (EM-clustering, and hierarchical clustering). The analysis showed that FC geological facies explained more of the variability in the environmental variables (Table S3). Therefore, Folk’s classification should be more applicable for delineating HZ facies at other locations.

The mechanisms of geological control on biogeochemical attributes might vary at locations with different sediment texture/structure and geomorphology. The samples analyzed in this study were collected over a relatively limited area close with coarser sediment texture (gravel dominated), and the variability in BGC properties among the local FC facies (G, MSG, MG, GM) is expected to be smaller in the study area than over larger stretches of the river that include sediments with a wider range of grain size distributions. Meanwhile, managed and seasonal river stage changes in the Columbia River also affect the water flow in the HZ and their related biogeochemical processes. The potential differences in biogeochemical activity will be examined in future studies that expand sampling at different seasons to locations with distinctly different hydrodynamic attributes (flow velocity, depth, shear stress) and geomorphological features (e.g., river bed form, bathymetric slope and aspect), where different sediment transport and depositional environment is expected to result in distinct sediment texture and BGC distributions.

Our results demonstrate strong relationships between microbial community assemblage structure, respiration, and grain size distribution. Previous work suggests that increased fraction of fine material such as mud reduces bed permeability and hence water residence time, which results in resource depletion - in particular oxygen - thus creating a distinct biogeochemical environment^13^. We observed negative correlations between the sediment characteristics %sand or %mud and organism modules, driven by near-universally negative relationships between individual OTUs and these characteristics. A high percentage of fine sediments can result in low pore connectivity and decreased hydraulic conductivity, and thus low recharge rates for O_2_ and organic carbon. Organisms we observed to be negatively correlated with %sand included highly abundant putative aerobic autotrophs (Thaumarchaea and Nitrospira). We noted only negative correlations with %mud which runs counter to the common assumption that sediments with higher concentrations of fines contain more organic carbon^77^. Relationships of soil respiration and organic C turnover to the size of microbial biomass are unclear^78,79^. In this study, we found that %TOC and biomass had inverse correlations with %sand and %mud, indicating that increased permeability, and thus decreased residence time, is critical to stimulating biological activity, likely by recharging electron donor and acceptor pools.

The high abundances of Thaumarchaea and Nitrospira suggest nitrification is an important process in the Columbia River hyporheic environment. Thaumarchaea, and Nitrospira, together comprised >20% of the community assemblage in most samples analyzed (Fig. 5). Interestingly, few ammonia-oxidizing bacterial taxa (AOB) were identified in these communities: only three OTUs were classified as Betaproteobacteria of family Nitrosomonadaceae with an average abundance of 0.6%. Similar results have been observed in marine environments. Waters in and around cold seeps in the ocean have been shown to have high abundance (20–60% of the microbial community), low diversity populations of Thaumarchaea^80^. In many environments, including soil, estuarine sediments, marine waters and sediments, freshwater lakes, and hot springs, AOA have been observed to outnumber AOB by orders of magnitude, although exceptions to this have also been reported (reviewed in ref.^81^). Investigations into what drives nitrifier community composition have implicated salinity, pH, and temperature^82^. In the Columbia River HZ, such factors are largely controlled by hydraulic regime, i.e. whether local conditions are groundwater- or river water-dominated. Spatial factors affecting exchange, such as sediment composition, should therefore influence the establishment and dynamics of these populations.

Other abundant (>0.5% average relative abundance) OTUs identified suggested a variety of other metabolisms. Several were related to aerobic heterotrophs such as the Actinobacterium *Gaiella*, the Rhizobiales *Variibacter*, and the Chthoniobacterales, members of which are capable of metabolizing plant-derived polysaccharides^83^. Others suggested anaerobic conditions exist within this generally oxidizing system, which might occur within restricted regions, e.g., in areas of the sediment dominated by fine-grained material; physical constraints on water movement influence residence time and hence O_2_ depletion in the HZ^84,85^. We found OTUs classified as a Chloroflexi of class Ardenticatenia, the described member of which is a facultative aerobic chemoheterotroph capable of growth via dissimilatory iron- and/or nitrate-reduction under anaerobic conditions^86^, Latescibacteria, which have previously been identified in anoxic environments^87^ and have many genes for the breakdown of algal-derived carbohydrates, and *Hyphomicrobium*, an Alphaproteobacterium for which the described species are facultative methylotrophic denitrifiers, capable of utilizing methanol, monomethylamine, or chloromethane^88,89^. The species richness across samples was 5–10x lower than reported for sediments sampled from the Tongue River^21^, which could be due to the lower sequencing effort in this study yielding a less comprehensive survey or differences in local environmental conditions selecting for a less diverse community.

Nitrification is thought to be an important function within the HZ, but the relationship between environmental conditions and ammonia-oxidizing organisms is complex. Studies targeting the ammonia oxidase (*amoA*) gene have examined the effect of salinity^90–94^ and nutrients^95–107^ on the relative abundances and activity of AOA and AOB but have yielded conflicting results. Other studies have indicated a seasonal cycling of AOA and AOB, suggesting C speciation or temperature may be drivers of population abundances. It is also possible that interactions with other organisms are critical, and a broader knowledge of the microbial community structure and function will be necessary to understand terrestrial and aquatic nitrification processes. The dearth of AOB in the Columbia River HZ may be related to the available NH_4_ pool that possibly vary with respect to gsd-based facies. AOB can tolerate higher concentrations of NH_4_ and NO_2_, but may also require higher concentrations, it also appears the AOA have an unusually high affinity for ammonia that gives them an advantage when concentrations are low^108,109^. The solid phase exchange capability of Hanford hyporheic sediments may account for a large pool of exchangeable NH_4_ that results in low aqueous concentrations (results not shown). Under such conditions, AOA would be predicted to outcompete AOB, resulting in the observed distribution.

River basins and reaches are geologically heterogeneous where some regions experience a high degree of hyporheic exchange over short time scales while others experience limited exchange with low fluxes of electron donors and acceptors, even over long periods of time^2,7^. Sediment permeability affects resource availability and drives changes in microbial abundance and assemblage composition. In turn, microbial function alters the chemistry of the system through metabolic activity and the hydrology through growth and extracellular matrix formation^110^. Associations between microbial/BGC features and facies properties provide qualitative constraints for hydro-biogeochemical simulation models of hyporheic zone function. For example, in our study area, the influence of hyporheic exchange rate seems to dominate the impact of surface area; therefore, regions with coarser grain material have greater biomass, more microbial activity, and potentially a shift towards autotrophic nitrification. These qualitative shifts need to be captured by simulation models; if the models do not predict these shifts, it suggests a need for model exploration and refinement. The associations can also provide some quantitative constraints, such as the organic carbon content of sediments and shifts in organic carbon across facies. Our study suggests a complex, yet predictable, dynamic between sediment properties, hydrogeochemistry, microbial community and microbially-mediated nutrient transformation along a short reach. To better understand the role the HZ plays in global biogeochemical cycles will require both broader sampling regimes to capture a wider diversity of sediment classes and investigations at increased spatial resolution to characterize microbial communities and function within and across geologic facies. This will help reduce uncertainty in larger scale characterization and modeling, whereby those facies-relevant microbial and biogeochemical properties can be mapped to larger scale features, or facies, defined by a combination of sediment texture and surface water flow.

## Electronic supplementary material

Supplementary Figures

Supplementary Tables S1 and S2

Supplementary Table S3
