# Supplementary material for: Geochemical and Microbial Community Attributes in Relation to Hyporheic Zone Geological Facies
Source: Sci Rep. 2017 Sep 20;7:12006. doi: 10.1038/s41598-017-12275-w (PMC5607297; doi:10.1038/s41598-017-12275-w)
Supplement: Supplementary file 1 — Supplementary Figures [file 41598_2017_12275_MOESM1_ESM.pdf]

Supplementary Figures

Geochemical and Microbial Community Attributes in Relation to Hyporheic Zone Geological Facies

Z Hou[1*], WC Nelson[2*], JC Stegen[2], CJ Murray[1], E Arntzen[1], AR Crump[2], DW Kennedy[2], MC Perkins[3], TD Scheibe[2,4], JK Fredrickson[2], JM Zachara[5]

[1]Energy and Environment Directorate, Pacific Northwest National Laboratory, Richland WA USA

[2]Environmental and Biological Sciences Directorate, Pacific Northwest National Laboratory, Richland WA USA

[3]Graphic Design, Pacific Northwest National Laboratory, Richland WA USA

[4]Environmental Molecular Sciences Laboratory, Pacific Northwest National Laboratory, Richland WA USA

[5]Physical and Computational Sciences Directorate, Pacific Northwest National Laboratory, Richland WA USA

[*] Z Hou and WC Nelson contributed equally to this work

Correspondence to zhangshuan.hou@pnnl.gov

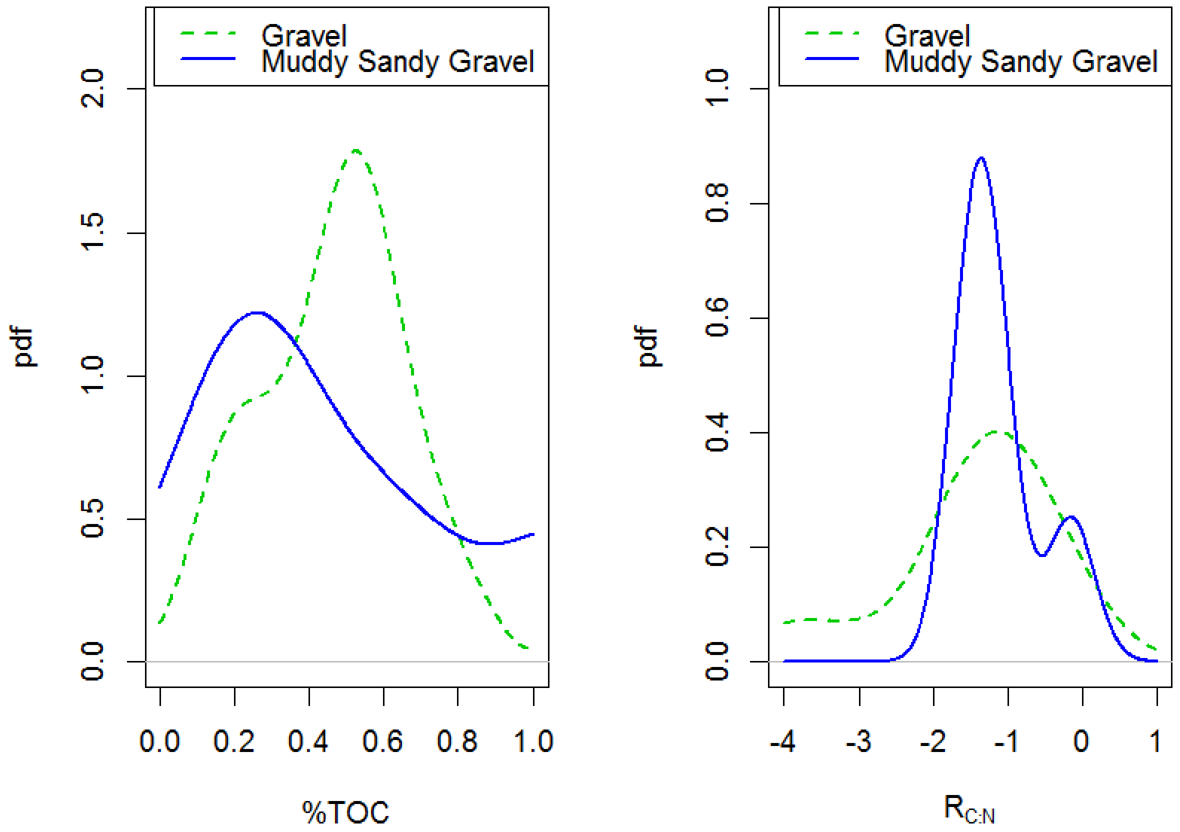

Figure S1. Statistical distributions of percent TOC and ratio of beta-glucosidase to N-acetyl-glucosaminidase and aminopeptidase activity ($R_{C:N}$) with respect to FC facies.

A

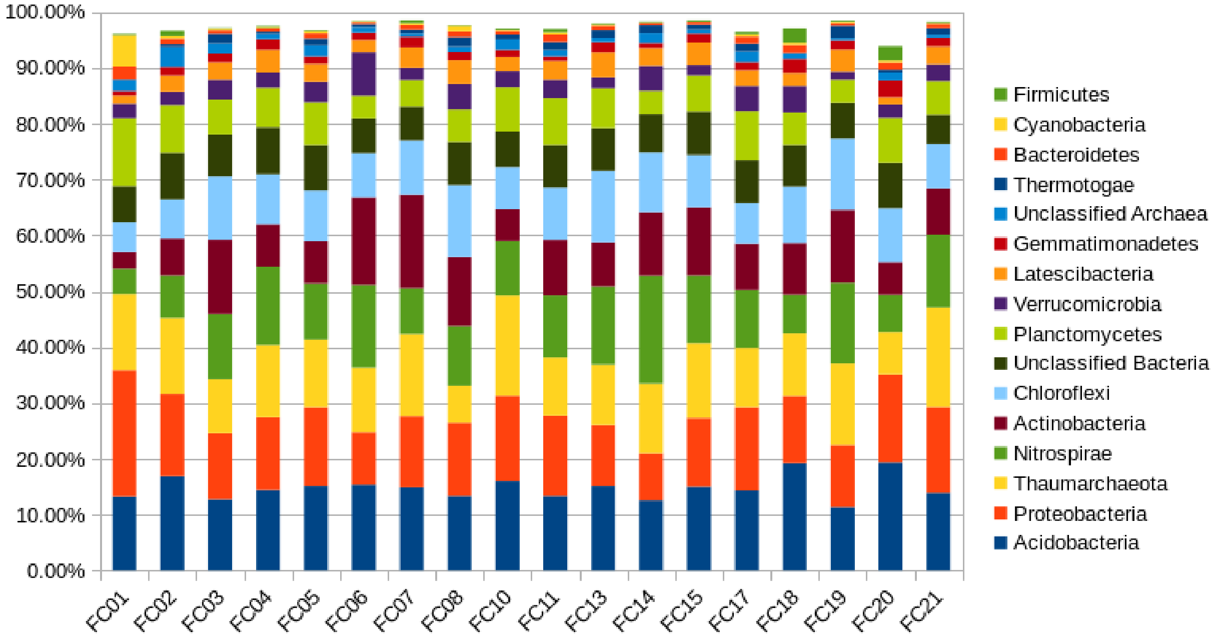

B

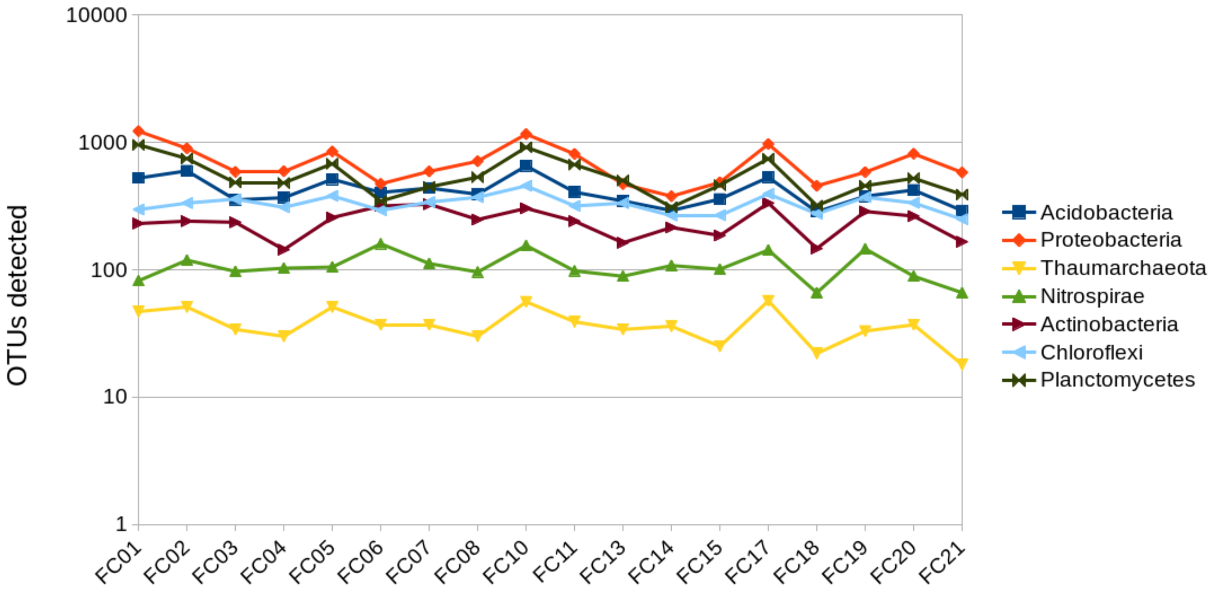

Figure S2. Diversity of hyporheic microbial communities. A) Phylum-level breakdown of OTUs. B) The number of distinct OTUs identified within abundant Phyla.

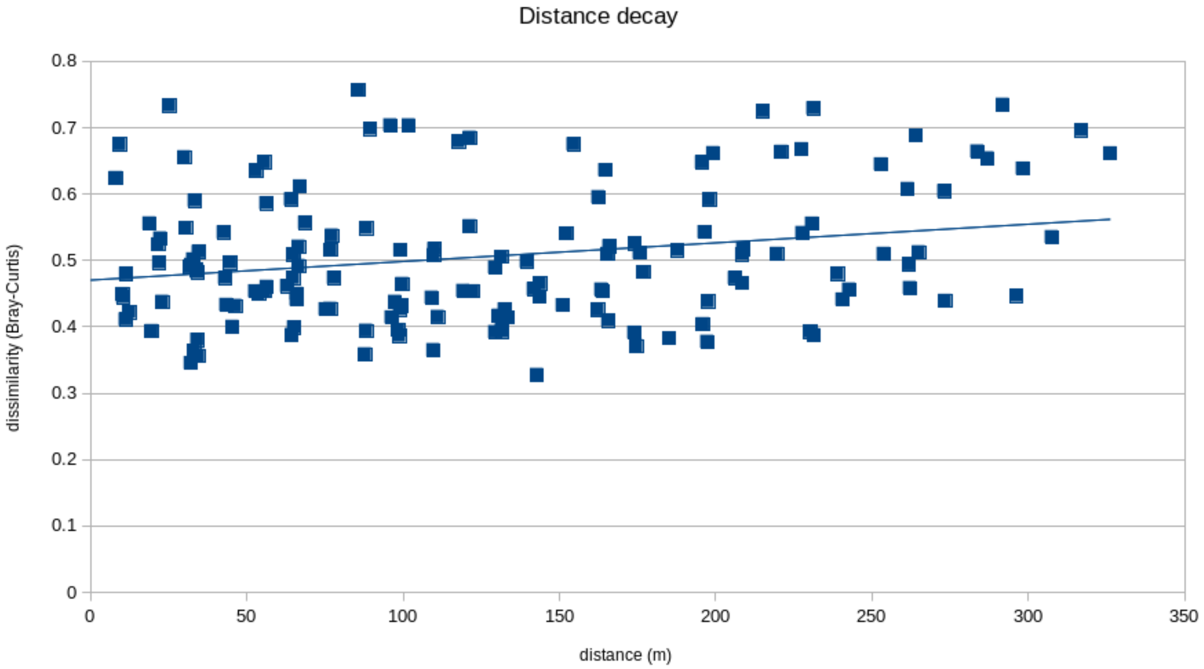

Figure S3 Decay of dissimilarity with distance. R2=0.0549

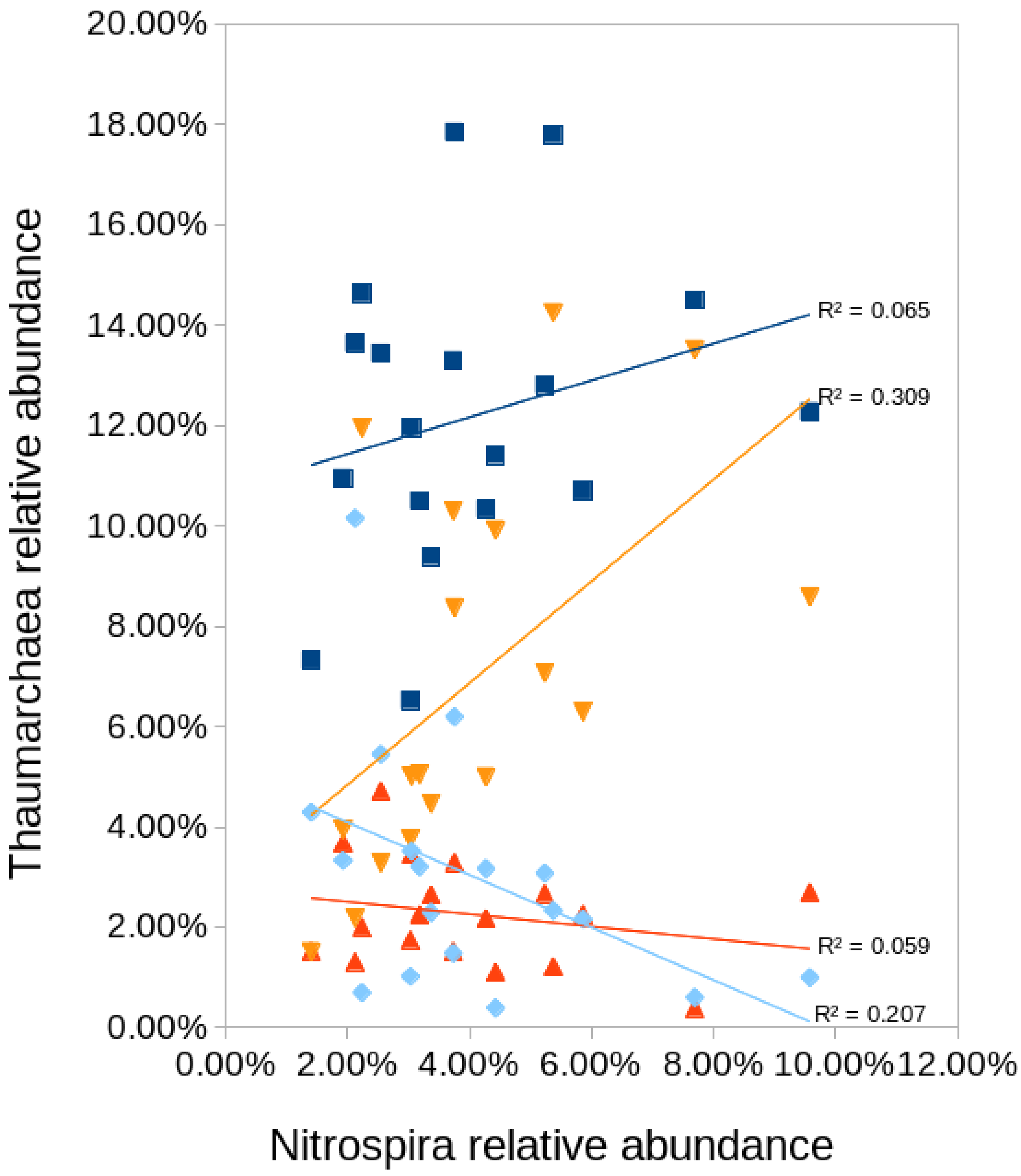

Figure S4. Correlation between Thaumarchaeal abundance and Nitrospira abundance. Dark blue squares, total Thaumarchaeal abundance; orange triangles, Soil Crenarchaeal Group (SCG); cyan diamonds, Marine Group I (MGI); red triangles, South African Gold Mine Crenarchaeal Group 1 (SAGMCG-1).
