# Supplementary material for: Geochemical and Microbial Community Attributes in Relation to Hyporheic Zone Geological Facies
Source: Sci Rep. 2017 Sep 20;7:12006. doi: 10.1038/s41598-017-12275-w (PMC5607297; doi:10.1038/s41598-017-12275-w)
Supplement: Supplementary file 3 — Supplementary Table S3 [file 41598_2017_12275_MOESM3_ESM.pdf]

Table S3. The variance of the selected environmental variables that can be explained by the FC, EM, and hierarchical classes. For all the tests, the degrees of freedom is 20 total and 17 residual, as there are four classes for each classification scheme.

	FC.facies	EM.Clustering	Hierarchical.Clustering
Ru	0.25	0.12	0.11
%TOC	0.19	0.12	0.01
C:N	0.35	0.22	0.11
$R_{C:N}$	0.12	0.02	0.18
$R_{C:P}$	0.17	0.01	0.10
$R_{N:P}$	0.10	0.07	0.08
